# Five new apterous species of the genus *Lathrobium* Gravenhorst (Coleoptera, Staphylinidae, Paederinae) from the Baishanzu Natural Reserve, East China

**DOI:** 10.3897/zookeys.251.3953

**Published:** 2012-12-18

**Authors:** Zhong Peng, Li-Zhen Li, Mei-Jun Zhao

**Affiliations:** 1 Department of Biology, College of Life and Environmental Sciences, Shanghai Normal University, Shanghai, 200234, P. R. China

**Keywords:** Coleoptera, Staphylinidae, taxonomy, *Lathrobium*, new species, Baishanzu, Zhejiang, China

## Abstract

Five new apterous species of the genus *Lathrobium* Gravenhorst, 1802 from Baishanzu Natural Reserve, Zhejiang, East China, *Lathrobium baishanzuense* **sp. n.**, *Lathrobium immanissimum* **sp. n.**, *Lathrobium obstipum* **sp. n.**, *Lathrobium pilosum* **sp. n.** and *Lathrobium tangi* **sp. n.**, are described and illustrated. The *Lathrobium* fauna of the study region is represented by two distinct lineages.

## Introduction

Up to date, 57 species of the genus *Lathrobium* Gravenhorst have been reported from mainland China, eleven of which are known from Zhejiang Province (Peng et al. 2012a, b; Watanabe 1999a, b; Watanabe and Luo 1992). Baishanzu is a nature reserve situated in Longquan and Qingyuan counties in southwestern Zhejiang province, eastern China. Medium mountain and hill areas constitute the main landform of the nature reserve and the dominant types of vegetation are evergreen broad-leaved forests and mixed coniferous and broad-leaved forests.

In 2004 and 2008, our colleagues conducted two expeditions to Baishanzu and collected numerous *Lathrobium* specimens from the floor of hardwood forest by sifting moist to wet leaf litter and humus (Tang pers. comm.).

Five apterous species were identified. Based on the morphology and chaetotaxy of the male sexual characters, they belong to two different species groups.

## Material and methods

The following abbreviations are used in the text, with all the measurements in millimeters:

BLlength of body from the anterior margin of the labrum to the apex of the abdomen;


FLlength of forebody from the anterior margin of the clypeus to the posterior margin of the elytra;


HLlength of head from the anterior margin of the clypeus to the posterior margin of the head;


HWmaximum width of head;


PLlength of pronotum along midline;


PWmaximum width of pronotum;


ELlength of elytra from the apex of the scutellum to the posterior margin of the elytra.


The type material is deposited in the Insect Collection of Shanghai Normal University, Shanghai, China (**SNUC**).

## Descriptions

### Lathrobium baishanzuense

Peng & Li sp. n.

urn:lsid:zoobank.org:act:EB3F420B-E491-45B8-B368-50433453191A

http://species-id.net/wiki/Lathrobium_baishanzuense

Figs 1A 3

#### Type material

(2 ♂♂)**.** Holotype: ♂, labeled ‘**CHINA:** Zhejiang Prov. / Qingyuan County / Baishanzu N. R. / 27°45’N, 119°13’E / 22–23.ix.2008, alt. 1,500 m / Tang & Zhang leg.’. Paratypes: 1 ♂, same label data as holotype, but ‘27°45'N, 119°12'E / 6.v.2004, alt. 1,400–1,700 m / Hu, Tang & Zhu leg.’.

#### Description. 

Measurements and ratios:BL 8.50–9.30, FL 3.00–3.63, HL 1.13–1.15, HW 1.26–1.31, PL 1.48–1.54, PW 1.30–1.35, EL 0.98–1.00, HL/HW 0.88–0.90, HW/PW 0.97, HL/PL 0.75–0.76, PL/PW 1.14, EL/PL 0.65–0.66.

**Figure 1.**
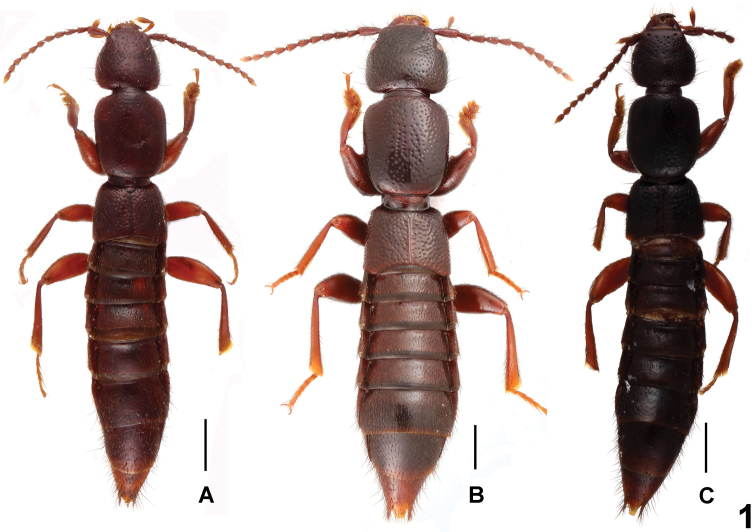
Habitus of *Lathrobium* spp., **A** *Lathrobium baishanzuense* **B** *Lathrobium immanissimum* **C** *Lathrobium pilosum*. Scale bars: 1.0 mm.

Habitus as in Fig. 1A. Body brown with paler apex, legs light brown, antennae brown to light brown.

Head subquadrate (HL/HW 0.88–0.90); punctation coarse and sparse; interstices with very shallow microreticulation; eyes small, approximately 1/4–1/3 the length of postocular region in dorsal view.

Pronotum nearly parallel-sided; punctation sparser than that of head; impunctate midline broad; interstices without microsculpture.

Elytra with punctation denser than that of pronotum and well defined; hind wings completely reduced.

Abdomen with dense punctation; interstices with very shallow, transversely striate microsculpture.

Male. Sternite V (Fig. 3A) with dark setae in postero-median impression and posterior margin with several point-like setae; sternite VI (Fig. 3B) with sparse, short setae in postero-median impression and posterior margin with 7–8 peg-like setae; posterior margin of sternite VII (Fig. 3C) weakly concave, pubescence unmodified; sternite VIII (Fig. 3D) with deep asymmetric emargination and blackish setae along both sides of this emargination; sternite IX (Fig. 3E) asymmetric; aedeagus (Fig. 3F, 3G) with asymmetric, apically truncate ventral process, short and moderately sclerotized dorsal plate, and without sclerotized spines in internal sac.

Female. Unknown.

#### Distribution.

East China: Donggong mountain range.

#### Etymology.

The specific epithet is derived from the type locality “Baishanzu”.

#### Comparative notes and comments.

Two distinct species groups occur in Baishanzu. One of these groups includes *Lathrobium baishanzuense*, the three following species from Baishanzu, and additionally *Lathrobium daicongchaoi* Peng & Li, 2012 from Guadun, *Lathrobium fujianense* Peng & Li, 2012 from Junzifeng Shan, *Lathrobium longwangshanense* Peng, Li & Zhao, 2012 from the Longwangshan, *Lathrobium tianmushanense* Watanabe, 1999 from the Tianmushan and the Longwangshan, *Lathrobium zhaotiexiongi* Peng & Li, 2012 from Jiulongshan Natural Reserve and Majian, and *Lathrobium jiulongshanense* Peng & Li, 2012 from Jiulongshan Natural Reserve. The species group is characterized by a male sternite V with a postero-median impression with dense dark setae and male sternites III–IV with conspicuously modified setae in the posterior or postero-median impression in some species, evident synapomorphies constituting the monophyly of this species group. These characters appear to be unique among Chinese *Lathrobium*.

Among the species of this group, *Lathrobium baishanzuense* is characterized particularly by the conspicuous modifications of the male sternite VIII and the morphology of the aedeagus.

**Figure 2.**
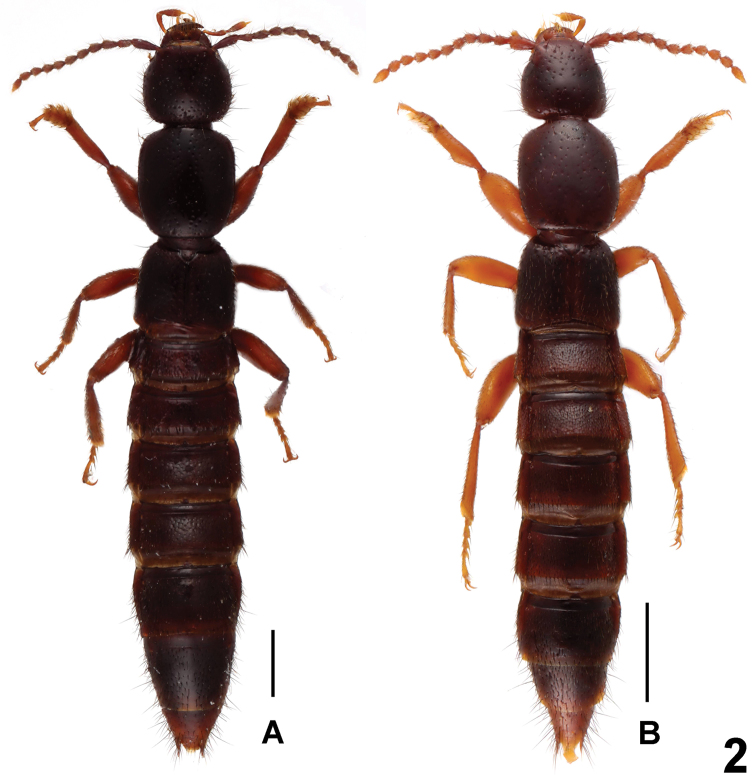
Habitus of *Lathrobium* spp., **A** *Lathrobium tangi* **B** *Lathrobium obstipum*. Scale bars: 1.0 mm.

**Figure 3.**
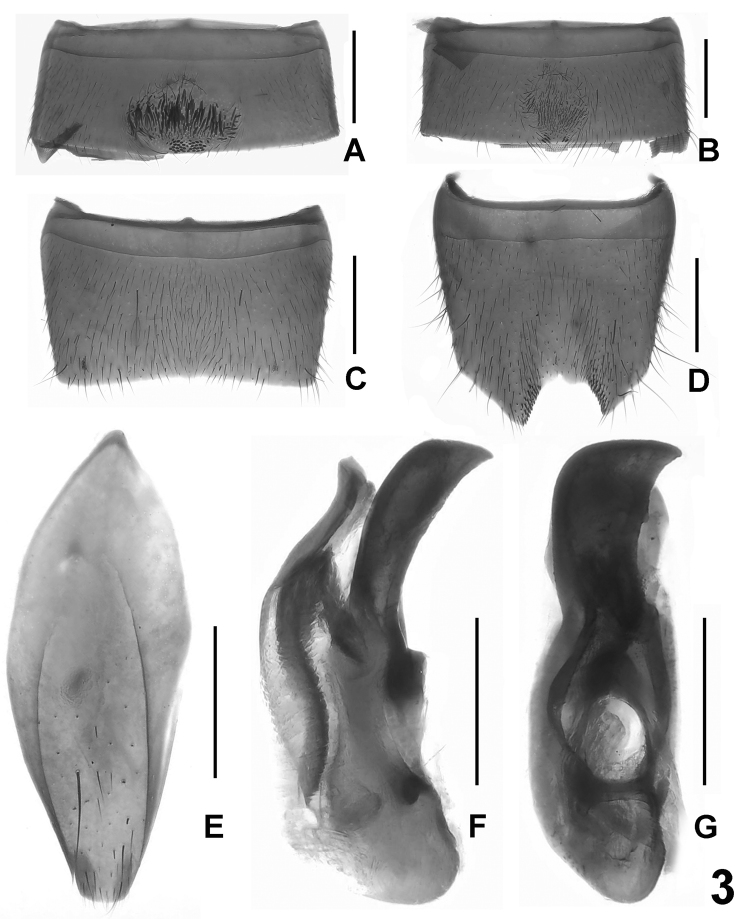
*Lathrobium baishanzuense*. **A** male sternite V **B** male sternite VI **C** male sternite VII**D** male sternite VIII **E** male sternite IX **F** aedeagus in lateral view **G** aedeagus in ventral view. Scale bars: 0.5 mm.

### Lathrobium manissimum

Peng & Li sp. n.

urn:lsid:zoobank.org:act:D23235A8-F1C1-447C-8F65-D1B46EDDDA8C

http://species-id.net/wiki/Lathrobium_immanissimum

Figs 1B 4

#### Type material

(5 ♂♂, 5 ♀♀)**.** Holotype: ♂, labeled ‘**CHINA:** Zhejiang Prov. / Qingyuan County / Baishanzu N. R. / 27°45'N, 119°13'E / 22–23.ix.2008, alt. 1,500 m / Tang & Zhang leg.’. Paratypes: 3 ♂♂, 4 ♀♀, same label data as holotype; 1 ♂, 1 ♀, same label data, but ‘27°44'N, 119°13'E / 21.viii.2004, alt. 1,250–1,650 m / Hu, Tang & Zhu leg.’.

#### Description.

Measurements and ratios:BL 10.57–12.51, FL 5.33–6.17, HL 1.63–1.70, HW 1.88–1.95, PL 2.23–2.30, PW 1.95–2.07, EL 1.40–1.50, HL/HW 0.86–0.88, HW/PW 0.94–0.96, HL/PL 0.73–0.74, PL/PW 1.11–1.14, EL/PL 0.63–0.65.

Habitus as in Fig. 1B. General appearance similar to that of *Lathrobium baishanzuense*, except for the much broader and larger body, the narrow impunctate midline on the pronotum and coarser punctation on the head, pronotum and elytra.

**Figure 4.**
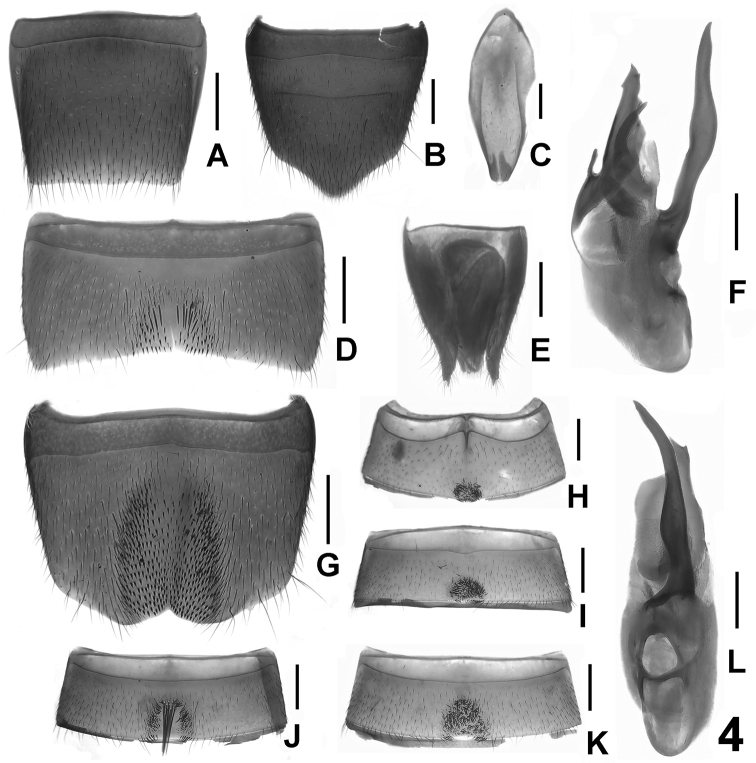
*Lathrobium immanissimum*. **A** female tergite VIII **B** female sternite VIII **C** male sternite IX **D** male sternite VII **E** female tergites IX-X **F** aedeagus in lateral view **G** male sternite VIII **H** male sternite III **I** male sternite IV **J** male sternite VI **K** male sternite V **L** aedeagus in ventral view. Scale bars: 0.5 mm.

Male. Sternite III (Fig. 4H) with dense dark setae in small posterior impression; sternite IV (Fig. 4I) with dense darkish setae in postero-median impression and posterior margin with several peg-like setae; sternite V (Fig. 4K) similar to sternite IV, but with sparser setae in larger impression; sternite VI (Fig. 4J) with long setae in postero-median impression and posterior margin with several peg-like setae; sternite VII (Fig. 4D) with sparse setae in postero-median impression; sternite VIII (Fig. 4G) with shallow symmetric emargination and short dark setae in large impression; sternite IX (Fig. 4C) asymmetric; aedeagus (Fig. 4F, 4L) with very long ventral process curved to the left in ventral view, broad dorsal plate and with single sclerotized apical spine in internal sac.

Female. Posterior margin of tergite VIII (Fig. 4A) truncate; sternite VIII (Fig. 4B) longer than that of male, posterior margin strongly convex; tergite X (Fig. 4E) obtuse apically and almost reaching anterior margin of tergite IX (Fig. 4E).

#### Distribution.

East China: Donggong mountain range.

#### Etymology.

The specific epithet (superlative of the Latin adjective immanis: huge) alludes to the size of the aedeagus.

#### Comparative notes

**.** The new species is readily distinguished from all eastern Chinese *Lathrobium* species by the large size and stout habitus, and by the presence of dense dark setae in the small posterior impression of sternite III.

### Lathrobium pilosum

Peng & Li sp. n.

urn:lsid:zoobank.org:act:547F958D-8C6F-4729-98A4-02038DE94A53

http://species-id.net/wiki/Lathrobium_pilosum

Figs 1C 5

#### Type material

(2 ♂♂)**.** Holotype: ♂, labeled ‘**CHINA:** Zhejiang Prov. / Qingyuan County / Baishanzu N. R. / 27°45'N, 119°13'E / 22–23.ix.2008, alt. 1,500 m / Tang & Zhang leg.’. Paratypes: 1 ♂, same label data as holotype, but ‘27°44'N, 119°13'E / 21.viii.2004, alt. 1,250–1,650 m / Hu, Tang & Zhu leg.’.

#### Description.

Measurements and ratios:BL 9.10–10.00, FL 4.11–4.17, HL 1.20–1.26, HW 1.39–1.50, PL 1.75–1.79, PW 1.52–1.57, EL 1.11–1.13, HL/HW 0.84–0.86, HW/PW 0.91–0.96, HL/PL 0.69–0.70, PL/PW 1.14–1.15, EL/PL 0.63.

Habitus as in Fig. 1C. Similar to *Lathrobium baishanzuense*, except for the darker coloration of body, the somewhat larger body size, coarser and sparser punctation on the head and pronotum, and weakly convex lateral margins of pronotum in dorsal view.

**Figure 5.**
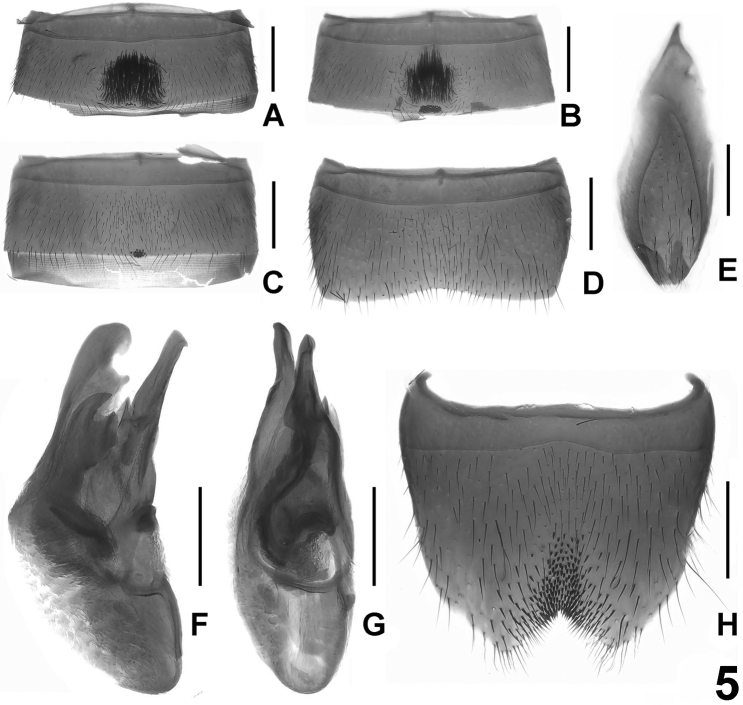
*Lathrobium pilosum*. **A** male sternite IV **B** male sternite V **C** male sternite VI **D** male sternite VII **E** male sternite IX **F** aedeagus in lateral view **G** aedeagus in ventral view **H** male sternite VIII. Scale bars: 0.5 mm.

Male. Sternite IV (Fig. 5A) with cluster of dense dark setae in large postero-median impression, and posterior margin with 7–9 point-like setae; sternite V (Fig. 5B) similar to sternite IV, but with longer setae in smaller impression and posterior margin with numerous point-like setae; tergite VII (Fig. 5D) strongly transverse, posterior margin weakly concave, pubescence unmodified; sternite VIII (Fig. 5H) with triangular, symmetric emargination and short darkish setae in narrow impression; sternite IX (Fig. 5E) nearly symmetric; aedeagus (Fig. 5F, 5G) with narrow ventral process, broad dorsal plate in lateral view and with single sclerotized apical spine in internal sac.

Female. Unknown.

#### Distribution.

East China: Donggong mountain range.

#### Etymology.

The specific epithet (Latin, adjective: hairy) alludes to the chaetotaxy of the male sternite IV.

#### Comparative notes.

This species is close to *Lathrobium tangi* sp. n. in sharing a large impression on the male sternite IV and a similar shape of the male sternite VII.In *Lathrobium pilosum*, the posterior margin of the male sternite VI has several point-like setae, and male sternite VIII is symmetrically emarginate.

### Lathrobium tangi

Peng & Li sp. n.

urn:lsid:zoobank.org:act:7AFC7FAB-C5C6-4837-8CE6-17B150F9B9D8

http://species-id.net/wiki/Lathrobium_tangi

Figs 2A 6

#### Type material

(3 ♂♂, 1 ♀)**.** Holotype: ♂, labeled ‘**CHINA:** Zhejiang Prov. / Qingyuan County / Baishanzu N. R. / 27°45'N, 119°14'E / 7.v.2008, alt. 1,050 m / Hu, Tang & Zhu leg.’. Paratypes: 1 ♂, same label data as holotype, but ‘22.ix.2008, alt. 1,200 m / Tang Liang leg.’; 1 ♂, same label data, but ‘28.vi.2004, alt. 1,100 m / Tang & Zhang leg.’; 1 ♀, same label data, but ‘27°45'N, 119°13'E / 22–23.ix.2008, alt. 1,500 m / Tang & Zhang leg.’.

#### Description.

Measurements and ratios:BL 8.62–10.06, FL 4.10–4.14, HL 1.11–1.20, HW 1.23–1.30, PL 1.65–1.70, PW 1.39–1.44, EL 1.04–1.11, HL/HW 0.90–0.92, HW/PW 0.88–0.90, HL/PL 0.67–0.71, PL/PW 1.18–1.19, EL/PL 0.63–0.65.

Habitus as in Fig. 2A. Similar to *Lathrobium baishanzuense*, except for the darker coloration of body, the somewhat larger body size, slightly coarser and sparser punctation on the head and pronotum, and weakly convex lateral margins of pronotum in dorsal view.

**Figure 6.**
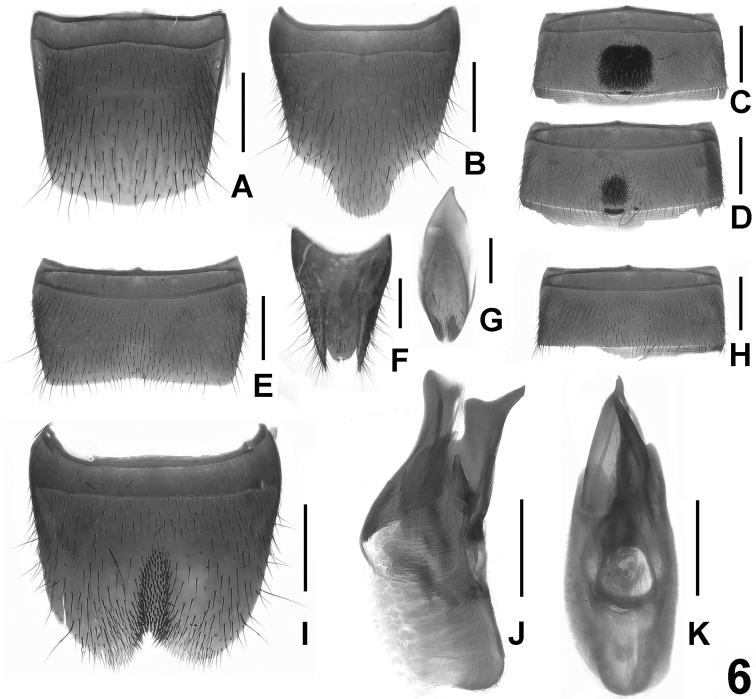
*Lathrobium tangi*. **A** female tergite VIII**B** female sternite VIII **C** male sternite IV **D** male sternite V **E** male sternite VII **F** female tergites IX-X **G** male sternite IX **H** male sternite VI **I** male sternite VIII **J** aedeagus in lateral view **K** aedeagus in ventral view. Scale bars: 0.5 mm.

Male. Sternite IV (Fig. 6C) with dense dark setae in large postero-median impression, and posterior margin with several point-like setae; sternite V (Fig. 6D) similar to sternite IV, but with much smaller impression, and posterior margin with numerous point-like setae; sternite VI (Fig. 6H) unmodified; posterior margin of sternite VII (Fig. 6E) weakly concave; sternite VIII (Fig. 6I) with subtriangular, asymmetric emargination and short dark setae in narrow impression; sternite IX (Fig. 6G) nearly symmetric; aedeagus (Fig. 6J, 6K) weakly asymmetric ventral process, large dorsal plate and with moderately sclerotized apical spine in internal sac.

Female. Posterior margin of tergite VIII (Fig. 6A) convex; sternite VIII (Fig. 6B) longer than that of male, middle of apical margin with apically convex projection; tergite X (Fig. 6F) convex apically, longer than tergite IX in the middle, but not reaching anterior margin of tergite IX (Fig. 6F).

#### Distribution.

East China: Donggong mountain range.

#### Etymology.

The species is named after Liang Tang, who collected the type seris.

#### Comparative notes.

*Lathrobium tangi* is similar to *Lathrobium pilosum* large impression on the male sternite IV and a similarlyshaped male sternite VII.In *Lathrobium tangi*, the posterior margin of the male sternite VI is unmodified and the male sternite VIII has an asymmetric emargination.

### Lathrobium obstipum

Peng & Li sp. n.

urn:lsid:zoobank.org:act:EA820283-AF9C-47BA-AB1F-AF0C34717C8E

http://species-id.net/wiki/Lathrobium_obstipum

Figs 2B 7

#### Type material

(17 ♂♂, 5 ♀♀)**.** Holotype: ♂, labeled ‘**CHINA:** Zhejiang Prov. / Qingyuan County / Baishanzu N. R. / 27°45'N, 119°13'E / 22–23.ix.2008, alt. 1,500 m / Tang & Zhang leg.’. Paratypes: 10 ♂♂, 5 ♀♀, same label data as holotype; 3 ♂♂, same label data, but ‘27°45'N, 119°12'E / 6.v.2004, alt. 1,400–1,700 m / Hu, Tang & Zhu leg.’; 3 ♂♂, same label data, but ‘27°44'N, 119°13'E / 21.viii.2004, alt. 1,250–1,650 m / Hu, Tang & Zhu leg.’.

#### Description.

Measurements and ratios:BL 6.34–7.23, FL 2.84–3.12, HL 0.65–0.71, HW 0.87–0.91, PL 1.11–1.19, PW 0.99–1.06, EL 0.76–0.81, HL/HW0.73–0.78, HW/PW 0.86–0.90, HL/PL 0.56–0.62, PL/PW 1.11–1.15, EL/PL 0.67–0.69.

Habitus as in Fig. 2B. Similar to *Lathrobium baishanzuense*, except for the lighter coloration of legs and antennae, the smaller body size, sparser punctation on the head and pronotum, and the somewhat broader impunctate midline on pronotum.

**Figure 7.**
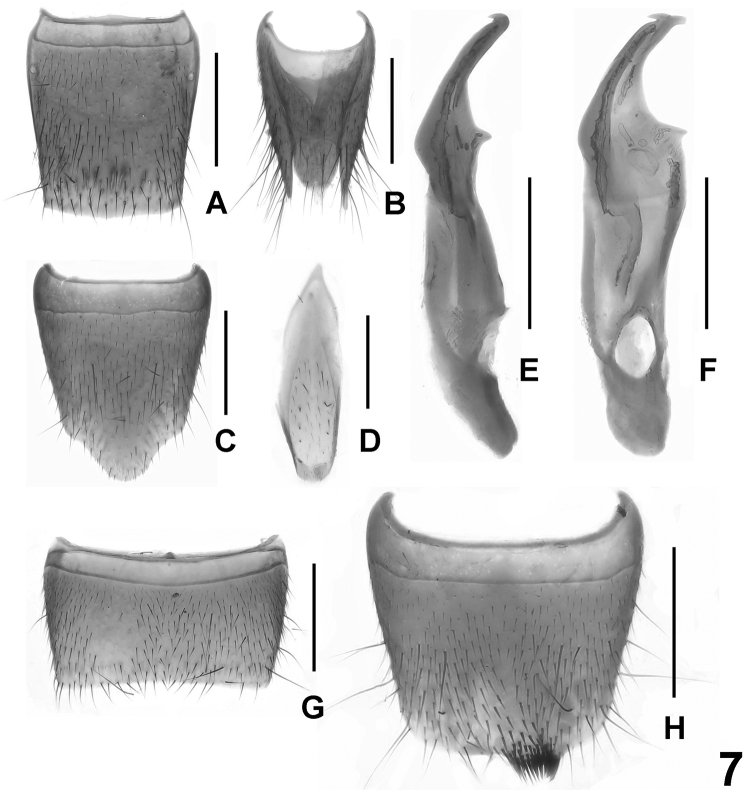
*Lathrobium obstipum*. **A** female tergite VIII **B** female tergites IX-X **C** female sternite VIII**D** male sternite IX **E** aedeagus in lateral view **F** aedeagus in ventral view **G** male sternite VII **H** male sternite VIII. Scale bars: 0.5 mm.

Male. Sternites III-VI unmodified; posterior margin of sternite VII (Fig. 7G) nearly truncate; posterior margin of sternite VIII (Fig. 7H) truncate and with tuft of short setae in asymmetric position; sternite IX (Fig. 7D) nearly symmetric; aedeagus (Fig. 7E, 7F) with asymmetric, apically forficate ventral process, without sclerotized dorsal plate, and without sclerotized spines in internal sac.

Female. Posterior margin of tergite VIII (Fig. 7A) weakly convex; sternite VIII (Fig. 7C) longer than that of male, posterior margin strongly convex; tergite X (Fig. 7B) convex apically, not reaching anterior margin of tergite IX (Fig. 7B).

#### Distribution.

East China: Donggong mountain range.

#### Etymology.

The specific epithet (Latin, adjective: awry) alludes to the chaetotaxy of the male sternite VIII.

#### Comparative notes and comments.

The new species is similar to *Lathrobium sheni* Peng & Li, 2012 from Jiulongshan in having similarly shaped male sternites VII and IX. The new species can be distinguished from *Lathrobium sheni* by a cluster of short setae on the posterior margin of the male sternite VIII and the distinctly flattened aedeagus. In *Lathrobium sheni*, the male sternite VIII has two rows of dense setae and the aedeagus is stout.

*Lathrobium obstipum* evidently represents a different lineage than the other species recorded from Baishanzu, since it does not share their derived modifications of the anterior male sternites. It is additionally distinguished from them by the smaller body, yellowish brown legs, the unmodified male sternite III-VI, the nearly truncate posterior margin of the male sternites VII and VIII, and the distinctly flattened aedeagus without a sclerotized dorsal plate.

## Supplementary Material

XML Treatment for Lathrobium baishanzuense

XML Treatment for Lathrobium manissimum

XML Treatment for Lathrobium pilosum

XML Treatment for Lathrobium tangi

XML Treatment for Lathrobium obstipum

## Erratum

Peng Z, Li LZ, Zhao MJ (2012) Two new apterous species of *Lathrobium* Gravenhorst (Coleoptera, Staphylinidae, Paederinae) from Fujian, East China. ZooKeys 218: 57–63. doi: 10.3897/zookeys.218.3361

The name *Lathrobium daicongchaoi* Peng & Li, 2012 occurs in the text in two different spelling variants: the intended and correct spelling is *Lathrobium daicongchaoi* (four times in the text on pages 58, 59, 60 and 61); the incorrect spelling is *Lathrobium daocongchaoi* (occurring once in the abstract on page 57).

## References

[B1] Peng Z Li LZ Zhao MJ (2012a) Taxonomic study on *Lathrobium* Gravenhorst (Coleoptera, Staphylinidae, Paederinae) from Longwangshan Mountain, East China. ZooKeys 165: 21–32 doi: 10.3897/zookeys.165.2384 22328854 10.3897/zookeys.165.2384 PMC3272631

[B2] Peng Z Li LZ Zhao MJ (2012b) Three new species of the genus *Lathrobium* Gravenhorst (Coleoptera: Staphylinidae: Paederinae) from the Jiulongshan Natural Reserve, East China. ZooKeys 184: 57-66 doi: 10.3897/zookeys.184.2634 22573952 10.3897/zookeys.184.2634 PMC3332015

[B3] Watanabe Y (1999a) Two new subterranean staphylinids (Coleoptera) from East China. Elytra 27 (1): 249-257

[B4] Watanabe Y (1999b) Two new species of the group of *Lathrobium pollens / brachypterum* (Coleoptera, Staphylinidae) from Zhejiang Province, East China. Elytra 27 (2): 573-580

[B5] Watanabe Y Luo ZY (1992) New species of genus *Lathrobium* (Coleoptera, Staphylinidae) from the Wu-yan-ling Natural Protective Area in Zhejiang. Elytra 20 (1): 47-56

